# Adverse Reactions to Antituberculosis Drugs in Iranian Tuberculosis Patients

**DOI:** 10.1155/2014/412893

**Published:** 2014-11-24

**Authors:** Aliasghar Farazi, Masoomeh Sofian, Mansoureh Jabbariasl, Sara Keshavarz

**Affiliations:** ^1^Department of Infectious Diseases and Tuberculosis and Pediatrics Infectious Diseases Research Center, School of Medicine, Arak University of Medical Sciences, Arak 3815934798, Iran; ^2^Department of Infectious Diseases, Valiasr Hospital, Arak University of Medical Sciences, Arak 3815934798, Iran; ^3^Department of Disease Control and Prevention, Health Center of Markazi Province, Arak University of Medical Sciences, Arak 3815934798, Iran; ^4^School of Medicine, Arak University of Medical Sciences, Arak 3815934798, Iran

## Abstract

*Background*. Antituberculosis multidrug regimens have been associated with increased incidence of adverse drug reactions (ADRs). This study aimed to determine the incidence and associated factors of ADRs due to antituberculosis therapy. *Methods*. This is a retrospective cross-sectional study on tuberculosis patients who were treated in tuberculosis clinics in Markazi province in Iran. The information contained in the medical files was extracted and entered into the questionnaire. Data was descriptively analyzed by using statistical package for social sciences (SPSS 18). *Results*. A total of 940 TB patients of 1240 patients' medical records available in 10 medical offices were included in this study. Of the 563 ADRs found in this study, 82.4% were considered minor reactions and 17.6% were major reactions. No death from antituberculosis ADR was observed. We found that the risk of major ADRs was higher in females (*P*  value = 0.0241), age >50 y (*P*  value = 0.0223), coinfection with HIV (*P*  value = 0.0323), smoking (*P*  value = 0.002), retreatment TB (*P*  value = 0.0203), and comorbidities (*P*  value = 0.0005). *Conclusions*. This study showed that severe side effects of anti-TB drugs are common in patients who have risk factors of ADRs and they should be followed up by close monitoring.

## 1. Introduction

Tuberculosis (TB) remains a major problem in health systems. In 2013, 6.1 million TB cases were reported to WHO and of these 5.7 million were people newly diagnosed and another 0.4 million were already on treatment. Incidence of tuberculosis in Iran was 21 (17–25) per 100000 people in 2013 [1]. More than 10000 tuberculosis (TB) patients are receiving directly observed treatment strategy (DOTS) in Iran every year. Single drug therapy can lead to the development of a bacterial population resistant to that drug. Inadequate treatment can lead to treatment failure, relapse, and drug resistance. Responsibility for successful treatment is assigned to the health care providers. First line antituberculosis drugs recommended by WHO are a combination of isoniazid, rifampicin, pyrazinamide, ethambutol, and streptomycin. It is important for clinicians to evaluate a patient's response to treatment to determine the efficacy of the treatment and to identify any adverse reactions. The adverse drug reactions may be mild to severe [2, 3]. Studies have shown that multidrug regimens can cause undesirable adverse drug reactions such as arthralgia, neurological disorders, gastrointestinal disorders, hepatotoxicity, and allergic reactions [4, 5]. ADRs increase patient discomfort and cause substantial additional costs because of excess outpatient visits, laboratory tests, and even in serious instances hospitalization [6]. In addition, ADRs are regarded as one of the major causes of nonadherence to anti-TB treatment [7]. At the same time, alternative drugs may cause severe complications with few effects. Adverse drug reactions may lead to prolonging of treatment, drug resistance, and treatment failure [8]. It may also increase morbidity and mortality of disease. The frequency, severity, and the nature of anti-TB therapy induced ADRs have been always a concern [9]. The overall incidence of ADRs caused by anti-TB therapy ranges from 5.1% to 83.5% [10]. We aimed to get an overview of ADRs due to anti-TB therapy and evaluate their impact on anti-TB treatment in Markazi province of Iran.

## 2. Methods

This is a retrospective cross-sectional study on tuberculosis patients who were treated in tuberculosis clinics in Markazi province in Iran. This study was approved by the Ethics Committee of Arak University of Medical Sciences. The population of study consisted of all tuberculosis cases treated at all counties (10 counties) in Markazi province via retrospective review of patient medical records from May 2010 to March 2014. Patient's medical records were sorted and selected according to the inclusion and exclusion criteria. Inclusion criteria for this study were as follows: patient diagnosed as having tuberculosis and taking first line anti-TB drugs regimen. Exclusion criteria of this study were as follows: patients with incomplete medical record and in comorbidity diseases the ADRs of organ with underlying disease excluded from analysis.

Adverse drug reaction was defined as a response which is noxious and unintended and which occurs at doses normally used in humans for the therapy of tuberculosis. An adverse drug reaction, contrary to an adverse event, is characterized by the suspicion of a causal relationship between the drug and the occurrence that is judged as being at least possibly related to treatment by the reporting or a reviewing health professional. For causality and severity assessment, all the suspected ADRs were discussed with the medical officer, treating clinician, and local specialist clinicians. Once a suspected ADR was identified, the clinicians were recorded and followed up until resolution or end of TB therapy. ADR patients modified their DOTS therapy and/or received symptomatic therapy according to the seriousness of the ADR. Follow-up was provided to all participants until the completion of DOTS therapy. Influence of various possible risk factors for developing ADRs was also studied. The causality was evaluated following the standards of WHO Uppsala Monitoring Center System; therefore, ADRs designated in this study were certain, probable, possible, unlikely, and unclassifiable. In the case of unlikely reactions, patients were classified as not experiencing an ADR [11].

Severity classifications were symptoms-based approach on the tuberculosis treatment guidelines, as mild reaction, in which there is no immediate modification of the standard regimen, and major reaction, which may entail interruption, dose reduction, drug replacement, and discontinuation of anti-TB drugs [12].

Liver dysfunction was defined as an increase in serum alanine aminotransferase (ALT), aspartate aminotransferase (AST), or total bilirubin higher than the upper limit normal (ULN) in two continuous tests but not considering the symptoms. Drug induced hepatitis was defined as increase of liver enzymes more than three times the ULN with the presence of hepatitis symptoms or increase to five times the ULN in the absence of symptoms and return to normal after withdrawal of all anti-TB drugs [13]. Hyperuricemia was defined as an increase in uric acid levels of more than 7 mg/dL. Anemia was defined as more than 1 g/dL drop in hemoglobin (Hgb) concentration after starting treatment. Neutropenia and thrombocytopenia were recognized as a drop in absolute neutrophil count and platelet count equal to or less than 1500 cells/mm^3^ and less than 150000 cell/mm^3^, respectively [14]. Liver dysfunction, hematologic system disorders, and renal impairment were determined based on laboratory examination, and other ADRs including gastrointestinal disorders, allergic reactions, arthralgia, and neurological disorders (auditory nerve damage, optic nerve damage, peripheral nerve damage, and central nervous system damage) were determined based on symptoms and physical examination. Collected data were analyzed using SPSS version 18.0. Categorical variables such as nationality, type of tuberculosis, patient's gender, and others were compared by Fisher's exact test and chi-square. Multiple logistic regression analysis was performed to identify the risk factors and the strength of association was measured by odds ratio (OR) with 95% confidence interval (95% CI) and a two-tailed *P* value of <0.05 was considered significant.

## 3. Results

A total of 1240 TB patients records were reviewed in this study and after the inclusion and exclusion criteria were applied, 940 cases remained eligible for the study. The records of 300 patients were excluded because of incomplete records. The age of patients ranged from 4 to 89 years (mean = 57.8 + 18.4 years; median = 59 years). Of the participants, 463 (49.3%) were male, 56.8 percent were urban, and 20.3 percent were immigrant (Afghans). Also 3.2 percent of patients were retreatment cases and 1.9 percent of patients were HIV^+^. In this study patients with pulmonary tuberculosis were 76.4% and 59.1% of the patients had more than one-month delay in diagnosis. The organ systems most affected by ADRs were the hepatobiliary system (35.7%), the gastrointestinal tract (22%), the musculoskeletal system (19.5%), the skin and appendages (15.3%), the peripheral nervous systems (3%), the hematologic system (1.2%), ototoxicity (1.2%), visual system (1.1%), and renal system (0.9%).  Table 1 describes the incidence, onset time, and seriousness of anti-TB ADRs. In the study sample, 94.4% of the reactions occurred during the first two months of treatment.

The frequency of smokers was 18.5% and most frequent comorbidities in the patients were lifestyle-related problems, hypertension, and cardiac diseases (10.8%), diabetes (8.4%), COPD, and occupational lung diseases (4.6%). In the study, the proportion of ADRs among women was more than men (55.8% versus 44.2%, *P*  value = 0.0251). Patients aged 50 years or older had higher proportion of ADRs than other age groups (54.3% versus 45.7%, *P*  value = 0.0048). We found that the incidence of major and minor reactions was 5.8% and 22.8%, in a total of 940 patients, and total complications due to anti-TB drugs were 563 events observed in 269 patients. Of the 269 patients with ADRs, 59 developed only one ADR, while 210 patients developed two or more ADRs during the study period. Of the 563 ADRs found in this study, 82.4% were considered minor reactions, and 17.6% were major reactions. No death from antituberculosis ADR was observed. We found that the risk of major ADRs was higher in females (*P*  value = 0.0241), age >50 y (*P*  value = 0.0223), coinfection with HIV (*P*  value = 0.0323), smoking (*P*  value = 0.002), retreatment TB (*P*  value = 0.0203), and comorbidities (*P*  value = 0.0005) (Table 2).

## 4. Discussion

The results of this study indicate that intolerance of anti-TB drugs due to the side effects is still a serious problem in patients with tuberculosis. In this study the incidence of severe side effects was the same as other studies (range 5.1–23%) [15]. Ayatollahi and Khavandegaran in Shiraz in Iran reported that 29.5% of the TB patients were with minor reactions and 5.2% with major reactions [16]. Taramian et al. in Gilan province in Iran reported that 27.3% of the TB patients were with more than one complication. Of the patients with complications, 48/2% were with hepatic dysfunction, 1/7% had ocular complications, 82% had gastrointestinal side effects, and 5/3 percent had skin side effects [17]. The incidence rate of hepatic dysfunction was found to be the most frequent side effect caused by anti-TB drugs in the present study. Arthralgia and arthritis with or without hyperuricemia were 3.6% in our study, although in another study nongouty arthralgia was observed in 17.6% of patients [18]. Also, it was accepted that the importance of arthralgia or drug induced hyperuricemia in the initial intensive phase of treatment was controversial [19, 20]. Pyrazinamide was discontinued due to persistent arthralgia with hyperuricemia in 3 (0.3%) patients in our study. Incidence of arthralgia resulted in the discontinuation of pyrazinamide similar to that reported in other series (0.2%, 2%) [15, 20]. Ototoxicity that manifested itself as either auditory or vestibular damage was found to be one of the severe side effects (0.4%) in the present study. All patients with ototoxicity used streptomycin for at least 3 weeks and two patients had used loop-inhibiting diuretic that was associated with an increased risk of ototoxicity [19], and streptomycin was discontinued immediately after the development of ototoxicity. While asymptomatic liver function disturbance was established in 181 patients (21.4%), rate of hepatitis was 4.3%. In some reports asymptomatic increase in serum liver enzymes occurs in nearly 20% to 30% of the patients with no need to alter treatment [21]. However, it must also be remembered that drug induced hepatitis is an important side effect that causes significant morbidity and mortality, and alteration of the drug regimen may be required [22]. It was determined that the incidence of hepatitis varied between 4.3% and 19% in published studies from various countries [20, 22–24]. For this reason, identification of risk factors of hepatitis is beneficial but despite the efforts spent for defining the exact predisposing factors of hepatotoxicity during anti-TB therapy predictors of developing hepatotoxicity are still controversial. In literature risk factors for hepatitis include high alcohol intake, female sex, older age, intake of other hepatotoxic drugs, poor nutritional status, preexisting liver disease, advanced disease, and acetylator status [20, 25]. Similar to multiple published studies, there was increased risk of hepatotoxicity among elderly patients in our study [15, 20, 22, 23, 25], although there are some studies in the literature, which have shown that there is no relationship between age and hepatotoxicity [26–28]. We found that the risk of major ADRs was higher (1.9 times) in female patients. There were some studies reporting a higher risk of ADRs in female patients [15, 20, 25–27] and also some other studies that show no differences between the two genders for developing ADRs [22, 26, 28, 29]. In our study smoking and Iranian nationality could be suggested as risk factors for major ADRs and also rifampin and isoniazid were the most causative agent for hepatotoxicity. The rate of severe peripheral neuropathy related to isoniazid was 0.3%, but Schaberg et al. found the rate of neurologic problems as 1.5% in their study [20], although it was higher in specific groups such as patients with chronic renal failure or multidrug resistance TB [30, 31]. In our study severe cutaneous adverse reaction rates with anti-TB drugs were 2.5%, lower than other studies (4.8% to 6%) [15, 20, 28, 32]. In this study, visual toxicity due to ethambutol occurred in 5 patients (0.5%) which was slightly higher than reported result in the study of Yee et al. (0.2%) [15]. Limitation of our study is that the results obtained are clearly not representative of all tuberculosis patients and the outcomes of all patients are not included because of deficient patient records. Another limitation is interactions between anti-TB drugs and other drugs in patients with comorbidity.

## 5. Conclusion

Good management of active tuberculosis treatment includes the initiation and the completion of anti-TB therapy with minimal complications. It must be kept in mind that severe side effects with anti-TB drugs are common especially among patients who have risk factors of ADRs and they should be followed up by closer monitoring for the ADRs related to anti-TB drugs. This study showed that 28.6% of TB patients who received standard treatment developed one or more ADRs that may result in increase in health care services and affect the anti-TB treatment pattern. Patients with ADRs were more susceptible to develop unfavorable results of anti-TB. This shows the importance of developing strategies to control ADRs both to improve the quality of life and to treat TB safely.

## Figures and Tables

**Table 1:** Incidence, onset time^*^, and seriousness of anti-TB adverse drug reactions.

Type of ADRs	Minor ADRs (%)	Major ADRs (%)	Mean of onset time (range) days
Gastrointestinal disorders	114 (91.9)	10 (8.1)	19 (4–57)
Liver dysfunction	162 (80.6)	39 (19.4)	17 (12–68)
Allergic reactions	63 (73.3)	23 (26.7)	20 (6–45)
Flu-like reactions	76 (100)	0	5 (1–9)
Arthralgia	29 (85.3)	5 (14.7)	36 (23–55)
Hematologic disorders	3 (42.8)	4 (57.2)	52 (29–81)
Peripheral neuropathy	14 (81.4)	3 (17.6)	48 (38–74)
Renal impairment	1 (20)	4 (80)	32 (27–41)
Visual impairment	1 (16.7)	5 (83.3)	46 (34–72)
Ototoxicity	1 (14.3)	6 (85.7)	33 (20–54)

Total	464 (82.4)	99 (17.6)	19 (4–81)

^*^It was from initiation of treatment. (It was the time that ADRs were found, not the exact time it happened.)

**Table 2:** Associated risk factors for major adverse drug reactions due to anti-TB drugs.

Variables	Without ADRs 671 (100%)	With major ADRs 54 (100%)	Adjusted odds ratio (95% CI)	*P* value
Age (years)				
≥50	296 (44.1)	33 (61.1)	1.99 (1.13–3.51)	0.0223
<50	375 (55.9)	21 (38.9)		
Gender				
Female	327 (47.8)	35 (64.8)	1.94 (1.09–3.46)	0.0241
Male	344 (51.3)	19 (35.2)		
Location				
Urban	382 (56.9)	30 (55.6)	0.94 (0.54–1.65)	0.8868
Rural	289 (43.1)	24 (44.4)		
Nationality				
Iranian	522 (77.8)	49 (90.7)	2.79 (1.10–7.15)	0.0362
Immigrant (Afghans)	149 (22.2)	5 (9.3)		
HIV status				
HIV+	7 (1.1)	3 (5.6)	5.58 (1.40–22.23)	0.0323
HIV−	664 (98.9)	51 (94.4)		
Smoking				
Smoker	112 (16.7)	19 (35.2)	2.71 (1.43–5.09)	0.002
Nonsmoker	559 (83.3)	35 (64.8)		
Addiction				
Addict	24 (3.6)	5 (9.3)	0.36 (0.13–0.99)	0.0569
Nonaddicts	647 (96.4)	49 (90.7)		
Diagnosis delay^*^				
≤30 days	269 (40.1)	23 (42.6)	0.90 (0.52–1.58)	0.7735
>30 days	402 (59.9)	31 (57.4)		
TB treatment history				
Primary	660 (98.4)	50 (92.6)	4.8 (1.48–15.62)	0.0203
Retreatment	11 (1.6)	4 (7.4)		
Type of TB				
Pulmonary	509 (75.9)	36 (66.7)	0.64 (0.35–1.15)	0.1416
Nonpulmonary	162 (24.1)	18 (33.3)		
Comorbidity (except HIV)				
Yes	160 (23.9)	25 (46.3)	2.75 (1.57–4.84)	0.0005
No	511 (76.1)	29 (53.7)		
Grading of sputum smear^**^				
<2+	129 (39.7)	11 (33.3)	0.76 (0.36–1.62)	0.5756
≥2+	196 (60.3)	22 (66.7)		

^*^Diagnostic delay: time interval between the onset of symptoms and labelling of the patient as a tuberculosis patient.

^**^WHO grading scale for AFB found by Ziehl-Neelsen smear microscopy:scanty, 1+, 2+, and 3+.
